# Voluntary Food Safety Disclosure and Government Grants: The Greater Food Approach Perspective

**DOI:** 10.1002/fsn3.70765

**Published:** 2025-08-03

**Authors:** Wentai Bi, Jie Huan, Peihang Zhang, Xudong Guo, Qi Qi, Yuan Liang

**Affiliations:** ^1^ College of Management Bohai University Jinzhou Liaoning P.R. China; ^2^ Party School of Liaoning Provincial Party Committee Shenyang Liaoning P.R. China

**Keywords:** food safety, government grants, market attention, the greater food approach, voluntary disclosure

## Abstract

Voluntary information disclosure is an important bridge between firms and external stakeholders. Based on the data of listed food companies from 2008 to 2022, this study examines the impact of voluntary food safety information disclosure on the government's resource allocation decisions from the perspective of the greater food approach. Such disclosure has a resource‐acquisition effect and can significantly increase the level of government grants. The aforementioned effect that is “not what it says on the tin” is only effective in the short term and does not allow for sustained benefits to be realized. The mechanism test established that the positive relationship between disclosure and government grants is significantly inhibited by market attention. Further research finds that this positive association is more pronounced among small‐scale and ordinary urban firms, with the types concentrated in revenue‐related government grants. This research enriches the literature on the economic consequences of voluntary disclosure and provides empirical evidence for the government to accurately screen firms' food safety status and information manipulation.

## Graphical abstract

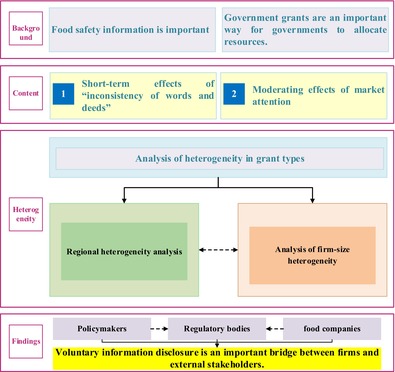

## 1 Introduction

The Food Information System covers the disclosure and sharing of information on the whole process of “from field to table,” which involves global issues such as food safety, industrial safety, and sustainable social development (Willett et al. 2019; Wanger et al. 2024; Zhang et al. 2024). The goal of the greater food approach highlights the process of transmutation of the information chain, sustainable response of the food chain, and concept of agri‐food system provisioning, while also putting forward higher requirements for global food safety information monitoring (Mu et al. 2024; Chiaraluce et al. 2024). Food firms are the microscopic subjects of food production and sales and are a key part of food safety governance (Hassoun et al. 2023). The ability to practice the information governance concept of the greater food approach is critically dependent on the quality of corporate food safety information disclosure and strategy selection. However, the information gap still exists in China, and the problem of “inconsistent” food safety information is still common (Li et al. 2024). For example, CCTV's “3–15” evening party exposed problems with product quality control at Shuanghui Group. The complexity and specialization of voluntary food safety disclosure strategies make them potential tools for corporate speculation or self‐interest risk. Furthermore, information asymmetry and market segmentation are exacerbated and the modernization of food safety risk governance and high‐quality development of the food industry hindered (Chen et al. 2020). Concurrently, government subsidies, as a scarce resource transferred to economic agents without compensation, are not universally available to all enterprises. Participation in policy‐oriented activities (e.g., precision poverty alleviation, charitable donations, etc.) and recognition by the government have been demonstrated to facilitate enterprises' acquisition of government resources (Du et al. 2019). In recent years, the construction of food safety has reached an unprecedented new level, and the strengthening of food safety supervision and the promotion of food safety governance have become the primary tasks of governments at all levels. The performance of enterprises' voluntary food safety information disclosure is related to a certain extent to the quality of local food safety, which will inevitably affect the government's evaluation of enterprises, and then affect the allocation of subsidies (Pawliczek et al. 2022). The optimal utilization of government subsidies, the promotion of voluntary food safety disclosure by enterprises, and the satisfaction of the public's demand for food safety and high‐quality consumption are pressing issues that China's practical and academic communities must address.

Government grants are an important way for governments to allocate resources. Firms' access to subsidies is not a random phenomenon and is affected by endowment characteristics, institutional biases, and other external circumstances (Huang 2022; Lago et al. 2024). Under the theory of promotion tournaments, government subsidies release a kind of “good signal,” thus carrying the function of policy guidance only in line with the national direction of enterprise activities to obtain the tilt of resource allocation and the “government certification” label (Guo et al. 2022). Meanwhile, as an important livelihood issue in China, local governments have to fulfill their food information regulatory responsibilities to maintain food safety effectively (Wu et al. 2024; Gao et al. 2023). Disclosure by firms is closely related to food safety status, which will inevitably signal food safety compliance to the government, and thus gain government support and resources. Whether food companies may take advantage of the lack of transparency and practicability of disclosure to whitewash the food safety situation or delay the disclosure of information to mislead the users of the information due to self‐interested motives, such as performance pressure and production regulation, is worth noting (Noh et al. 2019; Alhumud et al. 2025). This raises an interesting question: can governments detect “misrepresentation” manipulation in time to reduce government subsidies to food companies in the long run? That is, does manipulation of nonfinancial disclosure have only short‐term effects? Existing studies emphasize the importance of food safety disclosure, mostly in the context of government regulation of food safety systems. Compared to financial information, voluntary food safety information is more understandable and contains a large amount of information needed by the government, which facilitates the government's quick understanding of the firms and also reduces its information processing costs, which can help the government to identify the firms to be subsidized (Hassanein 2022; Elsayed and Hassanein 2024). However, the resource acquisition effect of voluntary information disclosure, especially based on the greater food approach perspective, has not been fully validated after the information interaction between supply and demand is completed, i.e., after firms have voluntarily disclosed food safety information and the government has completed its information search and screening. Accordingly, we discuss the following three issues: Firstly, does voluntary food safety disclosure enhance government grants in the context of the greater food approach? Secondly, does the resource‐acquisition effect of disclosure by firms that “misrepresent” themselves exist only in the short run? Thirdly, can noninstitutionalized regulation dampen the resource‐acquisition effect of disclosure?

The objective of this study is to examine the impact of voluntary food safety disclosure on government resource allocation decisions from a greater food approach perspective. An analysis was conducted on the data of listed food companies in China from 2008 to 2022. By doing so, we make three contributions to the literature. Firstly, the existing literature focuses on the manipulation of management tone, readability of corporate annual reports, and manipulation of financial information. However, the scenario of “inconsistency of words and deeds” caused by different types of voluntary disclosure strategies in real‐life situations is ignored. We explore the short‐term effects of “inconsistency” in disclosure by selecting the dimensions of actual behavior and theoretical strategies in annual reports, which is a new extension of voluntary disclosure behavior. Secondly, based on the new scenario of the greater food approach, we find that food firms have “inconsistent” food safety information performance. They disclose more theoretical food safety strategies to gain organizational legitimacy, which in turn shapes their corporate reputations to obtain government subsidies. In this regard, local governments need to support voluntary food safety information disclosure and, simultaneously improve the new mechanism of ex ante selection, ex ante supervision, and ex post evaluation of subsidy policies. Thirdly, noninstitutional market concerns can compensate for the shortcomings of institutional regulation, which is important for information governance effects and provides new perspectives for better subsequent noninstitutional regulatory practices.

## 2 Literature Review and Hypotheses Development

### 2.1 Voluntary Food Safety Disclosure and Government Grants

In the Chinese context, enterprise development cannot be separated from policy guidance and government support. Government grants are an important means for the government to allocate resources, and local governments usually grant them based on regional economic conditions, sustainable development goals, and corporate social responsibility (Wilson 1999; Lago‐Penas 2008). Local governments have discretionary power in selecting the firms to be allocated grants. To obtain positive support from the government and respond to policy guidance (such as agricultural poverty alleviation and social welfare activities), firms will adjust their own business strategies to obtain cooperation projects, tax incentives, and government grants (Zulu‐Chisanga et al. 2021; Edeh and Prévot 2024). Therefore, firms that actively disclose voluntary food safety information have a higher probability of obtaining government subsidies and key resources. The reasons for this may be twofold:

First, the “tournament competition” is a classic theoretical framework for explaining the behavior of Chinese governments at all levels (Tufail et al. 2023; Li et al. 2019). With the central government's increased attention on food safety, the local performance appraisal system indicators have become more diversified, shifting from “single economic growth” to “high‐quality construction of people's livelihoods and welfare (Lin, Xu, et al. 2024; Lin, Ma, et al. 2024).” In 2016, the State Council issued the “Opinions on Implementing the New Concept of Development and Accelerating the Modernization of Agriculture to Achieve the Goal of Comprehensive Well‐being,” which highlights that strengthening the accountability system for food safety is an important indicator for assessing the performance of party and government leadership. In 2023, the State Administration of Market Supervision issued and implemented the Interim Measures for the Supervision and Administration of Food‐Related Product Quality and Safety to urge firms to implement the main responsibility for food safety and strengthen the supervisory responsibility of local supervisors. Specifically, China's food safety governance has shifted from a “supervisory‐enterprise relationship” (an end‐to‐end control model based on the principle of “who produces, who is responsible”) to a “supervisory‐political relationship” (requiring the party and government to share responsibility for food safety) (Lin, Xu, et al. 2024; Lin, Ma, et al. 2024; Jin et al. 2024). The targeted performance of food safety supervision has had an increasingly significant impact at the local level.

At the same time, the decentralized financial system has strengthened the autonomy of local governments, which has led to the increasing prominence of local financial pressures to a certain extent, ultimately resulting in the formation of a certain “expenditure bias” in local finances (Nickell and Nicolitsas 1999). Food safety information governance is a long‐term, systematic, and complex project, and firms, as the first responsible body of food information, must crucially guide their behavior correctly (Li et al. 2024; Yadav et al. 2024). Thus, firms with high‐quality disclosure are more likely to be favored by the government and thus enjoy more government grants.

Second, resource dependence theory provides the theoretical support for elaborating the interactive relationship between government and firms. Firms acquire resources through interaction with the external environment and reshape their roles in the external dependence of resource exchange (Yao et al. 2023; Schmid 2004). The sustained operation of firms cannot be separated from the allocation and support of scarce public resources, such as government subsidies, which are crucial to the high‐quality development of firms. From a practical viewpoint, food firms have proactively assumed responsibility for food safety disclosure and cooperated with the government's food safety governance activities, which helps secure new competitive advantages for enterprise development (Chen et al. 2020). With the concept of food safety taking root in people's minds, disclosure not only helps food safety regulation and sends quality signals to the market, but also indirectly helps firms obtain more subsidized resources. Summarizing the discussion above, we formulate our first hypothesis:

Hypothesis 1 *Voluntary food safety disclosure contributes significantly to government grants*.

### 2.2 The Short‐Term Effects of Voluntary Food Safety Disclosures That “Don't Mean What They Say”

Signaling and legitimacy theories explain the motivation for differences in disclosure from different theoretical perspectives (Friske et al. 2023; Connelly et al. 2024). Compared to external stakeholders, a firm's internal information advantage is obvious, and opportunistic motives may drive it to abuse its information advantage to signal quality value to the outside world and bring more resources (Kim and Valentine 2023). However, China currently lacks a standardized and unified framework for voluntary food safety information reporting. Firms are more likely to disclose noncompliant food safety information to whitewash their food safety production status, and cases of hiding bad news, low transparency of information, and selective disclosure may exist (Wang et al. 2023). The quantity and quality of information disclosure are mainly measured by report length and text professionalism, credibility, and understandability. Poor performers gain market credibility for their companies by disclosing more qualitative information, making superficial statements using word games, and using vague language (Zhu et al. 2019). From a signaling perspective, “inconsistent” disclosure reduces the accuracy of external identification of corporate information. Such disclosure serves a warm‐up or strategic purpose, ultimately creating a false sense of corporate goodwill and promoting corporate reputation and value (Pope et al. 2024). From the perspective of legitimacy, the firm's annual report is the primary source of legitimacy performance. At the same time, food safety information, as a voluntary information disclosure, has a strong “self‐service” motive. A “justification” behavior of legitimacy management exists (Roszkowska‐Menkes et al. 2024; Akhter et al. 2023).

Information asymmetry allows companies to manipulate information for personal gain; however, over time, the true state of affairs emerges and is eventually exposed to the outside world (Romito and Vurro 2021). Even if enterprises take advantage of the information to avoid regulation in the short term, they adopt the disclosure strategy of “inconsistent” to obtain government subsidies and other key resources. However, in the medium to long term, the actual state of food safety will gradually emerge as a result of food safety regulators', investment institutions', consumers', and other stakeholders' attention and data mining. As the transparency of information increases, the government's information screening ability and regulatory intensity will be adjusted accordingly, and the resource‐acquisition effect of “inconsistent” disclosure will gradually disappear (Mandal and Jain 2021). Thus, we derive our second hypothesis as follows:

Hypothesis 2 *The resource‐acquisition effect of voluntary food safety disclosure of “inconsistents” is only effective in the short term*.

**FIGURE 1:**
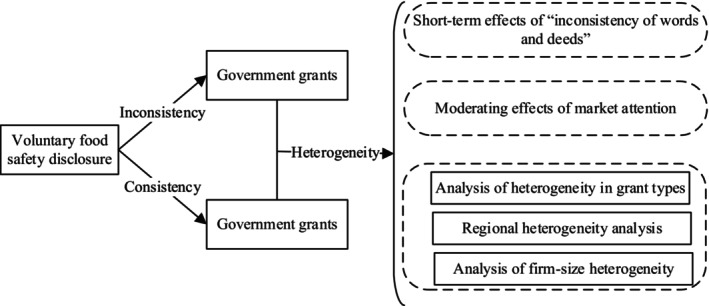
The conceptual framework.

Figure 1 provides the fundamental analysis logic: the impact of voluntary food safety disclosures on government grants.

## 3 Data and Methodologies

### 3.1 Data Sources

This study constructs panel data of food firms to analyze the impact of disclosure on government grants. The A‐share listed food companies in Shanghai and Shenzhen from 2008 to 2022 are used as the initial sample. We choose 2008 as the starting point of the study because A‐share listed companies only started to disclose social responsibility reports routinely, one after another, in this year. On this basis, the samples are further processed as follows: (1) samples with missing values and extreme outliers for key variables are eliminated; (2) all continuous variables are shrink‐tailed by 1% up or down; and (3) samples with less than 2 years of listing are deleted. Finally, 205 listed companies with a total of 2048 valid observation samples are obtained. The data related to disclosure are obtained from food companies' annual and social responsibility reports collected and organized manually. The data for the remaining variables are obtained from the China Economic and Financial Research Database (CSMAR).

### 3.2 Variables and Their Measurement

#### 3.2.1 Dependent Variable: Government Grants

The dependent variable is government grants (*Sub*). The total value of financial subsidies received by listed companies from government grants is reflected in the item “Subsidy Income” in the income statement. The detailed account of subsidy income is disclosed in the notes to the financial statements. Therefore, the present study manually collects and organizes the data of government grants received by listed companies. This is done based on the database of financial statement notes in the CSMAR database. Referring to the practice of related studies (Dan and Ling 2018), the natural logarithm of the subsidies received by firms in the year is used to measure the level of government grants (*Sub*_1_). That of the actual amount of grants received by sample firms in the year is used to measure the intensity of government grants by taking the natural logarithm of the ratio of the actual amount of grants received in the year to the average of the grants received by firms in the year of the industry they belong to after adding 1 (*Sub*_2_).

#### 3.2.2 Independent Variable: Voluntary Food Safety Information Disclosure

**FIGURE 2:**
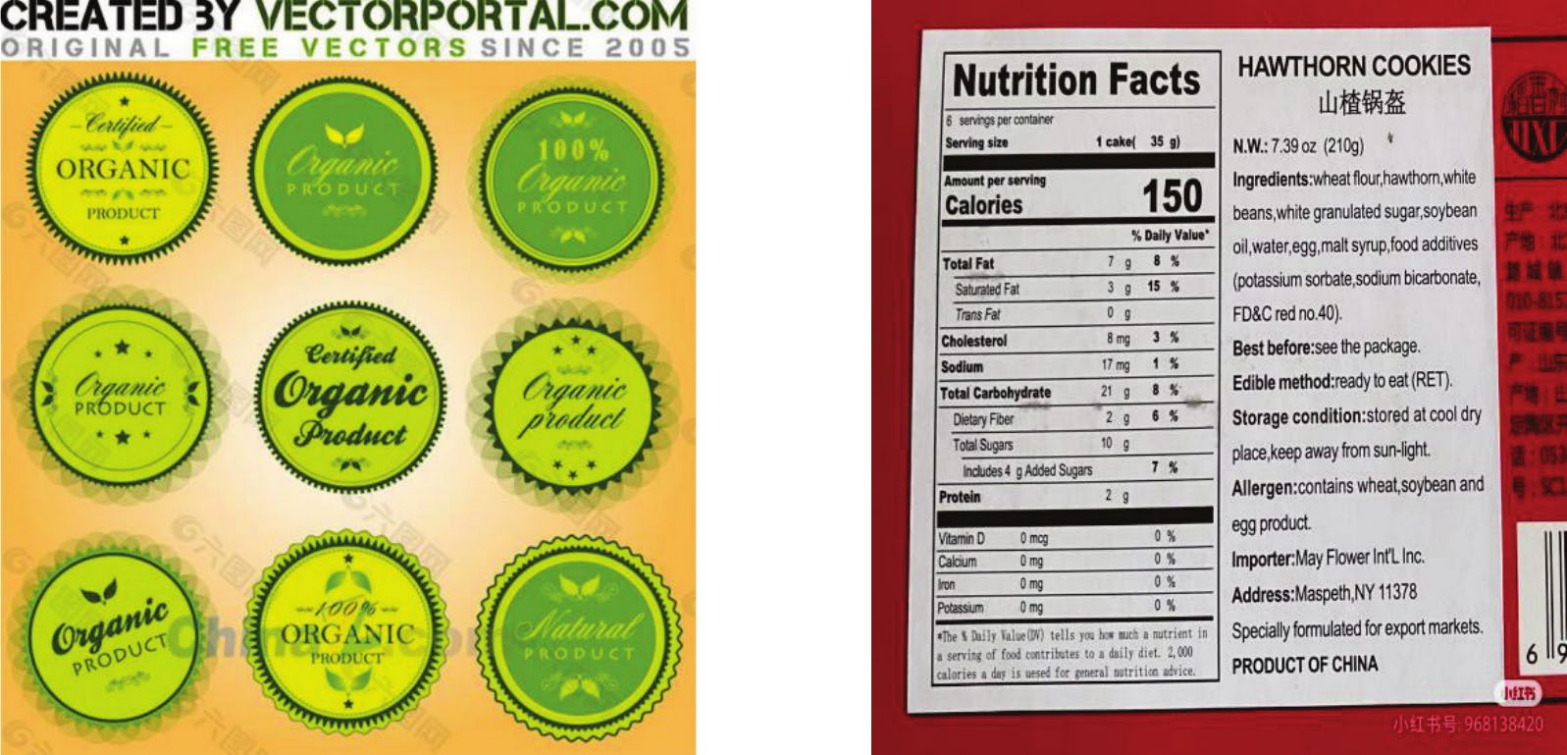
Mandatory food labeling.

The independent variable is voluntary food safety information disclosure (*Fsid*). A key research design issue is to develop a comprehensive and reliable indicator system for such disclosure. As demonstrated in Figure 2, numerous countries currently mandate the mandatory disclosure of food safety information by companies in product labeling. This includes information such as the date of production, the place of production, and a list of the product's nutritional content. In contrast, the present study focuses on voluntary food safety information provided in corporate annual reports and social responsibility reports. The voluntary disclosure of food safety information can be accomplished in a variety of ways, including the creation of a standalone report, its incorporation into the annual report, or its publication on the company website. The information can be financial or nonfinancial, quantitative or qualitative. Referring to Clarkson et al. (2008), environmental disclosure rating scale (an evaluation system that highlights the focus of investors and analysts, has comprehensive coverage, and is considered the most authoritative evaluation in the field of disclosure), the content analysis method is utilized to construct evaluation indicators for disclosure. The specific method is to search “traceability,” “product quality,” “ISO9001 certification,” “risk management,” “food safety disclosure” and “food safety disclosure” from the annual reports of the sample companies, taking the content of food companies' disclosure into account. The key words “risk management” and “food safety” from the sample enterprises' annual reports, as well as the annual and social responsibility reports are used to collect food enterprises' disclosure information (Chen et al. 2020; Bi et al. 2024). The selected observations cover key aspects of food safety, including corporate food safety policies, production and distribution areas, quality and safety controls, and internal industry evaluations.

Strategic Concept (*Stra*) is considered a “soft” disclosure item, and consists of two primary and seven secondary indicators: Vision and Strategy Statement and Business Image; Actual Behavior (*Beha*) is considered a “hard” disclosure item, which is difficult to be imitated by poorly performing companies in terms of food safety and security. *Beha* is considered a “hard” disclosure item that is difficult to be easily imitated by companies with poor food safety performance. The item mainly consists of four level 1 indicators and 12 level 2 indicators for governance structure and management, control of suppliers and sellers, control of production process, and control of employee behavior. One point is assigned for each disclosure, and enterprises that disclose both the substance of the information and detailed values (e.g., 99.35% pass rate for physical quality of products) are given an additional one point for a total of two points. The collection of both strategic conceptual data and actual behavioral data was conducted manually from the annual reports of various firms. Points were scored item by item according to the actual situation of food firms' disclosure and summed up to obtain the voluntary food safety disclosure score (Appendix 1). To ensure the accuracy of the study data, 100 samples were randomly selected for manual review, and the rules were explained to the scorers before the formal review to help them understand the assessment process and standards. The review results indicated that the consistency of the scores was in line with the objective reality and needs of the study, which ensured the objectivity and accuracy of the results.

#### 3.2.3 Moderating Variables: Market Attention

The moderating variable is Market Attention (*Ma*), which is composed of Research Reported Attention (*Ra*) and Analyst Attention (*Aa*). The exportation of data for these two variables can be performed directly from the database. The researched report attention variable is measured using the number of research reports tracking and analyzing firms +1, taking the natural logarithm; the analyst attention variable is measured using the number of analysts tracking +1, taking the natural logarithm.

#### 3.2.4 Control Variables

A comprehensive review of pertinent studies on corporate behavior is conducted, along with the consideration of additional factors that may influence information disclosure and government grants. This approach is undertaken to ensure the scientific and precise outcomes of the study for the selection of control variables. In order to minimize the impact of omitted variable bias, two types of variables, namely, relevant financial indicators and corporate governance indicators, are mainly controlled for with reference to past literature (Li et al. 2024; Dan and Ling 2018). Among them, the financial indicators include firm size (*Size*), leverage ratio (*Lev*), return on assets (*Roa*), revenue growth rate (*Growth*), and operating cash flow (*Cash*). The corporate governance indicators include board size (*Board*), dual position (*Dual*), shareholding concentration (*Share*), proportion of independent directors (*Ratio*), fixed number of years (*Age*), nature of ownership (*Soe*), and executive remuneration (*Salary*). Appendix 2 tabulates the variable definitions and their measurement.

### 3.3 Econometric Models

The fixed effects model is a common approach when dealing with panel data. Voluntary food safety disclosure involves enterprises in different regions, each of which may have its own unique characteristics and stage of development. The fixed effects model allows controlling for the individual effects of these enterprises through the introduction of dummy variables. This helps deal with individual heterogeneity and thus estimate the effects of other explanatory variables on government grants more accurately. Consequently, the fixed effects model is regarded as the optimal approach. In order to test the previous hypotheses, this study uses the following benchmark model to examine the impact of voluntary food safety disclosure on government grants:

(1) $${\mathrm{Sub}}_{\mathrm{it}}=\beta_{0}+\beta_{1}{\text{Fsid}}_{\mathrm{it}}+\beta_{2}{\text{Controls}}_{\mathrm{it}}+\gamma_{m}+\gamma_{t}+\varepsilon_{\mathrm{it}}$$

Where ${\text{Fsid}}_{i,t}$ is voluntary food safety disclosure by firms. ${\mathrm{Sub}}_{i,t}$ is the government subsidies received by firms, and Sub1 and ${\mathrm{Sub}}_{2}$ are constructed to further discuss the effects of the level and intensity of government subsidies. Focus on the sign and significance of the coefficient of ${\mathrm{Sub}}_{i,t}$, whose economic implication is the effect of voluntary food safety disclosure on government subsidies. ${\text{Control}}_{i,t}$ is the control variable. $\varepsilon_{i,t}$ is the random error term. $\gamma_{m}$ and $\gamma_{t}$ denote area fixed effects and time fixed effects.

To further verify the moderating effect of market attention on the relationship between voluntary food safety disclosure and government grants, the following moderating effect model is constructed on the basis of the baseline model using the attention of the researched newspaper and the attention of the analysts as proxy variables:

(2) $${\mathrm{Sub}}_{\mathrm{it}}=\beta_{0}+\beta_{1}{\text{Fsid}}_{\mathrm{it}}+\beta_{2}{\text{Controls}}_{\mathrm{it}}+\beta_{3}{\mathrm{Ma}}_{\mathrm{it}}+\beta_{4}{\mathrm{Ma}}_{\mathrm{it}}\times {\text{Fsid}}_{\mathrm{it}}+\gamma_{m}+\gamma_{t}+\varepsilon_{\mathrm{it}}$$

${\mathrm{Ma}}_{\mathrm{it}}$ is the market concern, specifically the researched report concern (*Ra*) and analyst concern (*Aa*). ${\mathrm{Ma}}_{\mathrm{it}}\times {\text{Fsid}}_{\mathrm{it}}$ represents the interaction item between market attention and voluntary food safety disclosure.

## 4 Results and Discussion

### 4.1 Descriptive Statistics

Descriptive statistics for the main variables are presented in Table 1. Because the government grants variable is standardized, these statistics only reflect the relative size of those received by enterprises. The amount of grants varies among firms, and they do not have the same level of grants. The standard deviation and mean of disclosure are 2.868 and 10.217, respectively, thus indicating that a significant gap exists in the quality of disclosure among different listed enterprises. The mean value of firm size in the sample is 21.963, the proportion of state‐owned enterprises is 39.4%, the ratio of cash flow from operating activities to total assets is 6.9%, and the mean value of dual position is 30.2%, thereby indicating that approximately 30% of the firms have a concurrent chairperson and general manager. The descriptive statistics of the main variables are basically consistent with previous literature and will not be repeated.

**TABLE 1:** Descriptive statistics.

Variable	*N*	Mean	SD	Min	p50	Max
*Sub*_1_	2048	15.541	3.216	0.000	16.104	21.086
*Sub*_2_	2048	0.864	1.695	0.000	0.304	17.660
*Fsid*	2048	10.217	2.868	1.000	10.000	18.000
*Stra*	2048	3.999	1.432	0.000	4.000	7.000
*Beha*	2048	5.888	2.095	0.000	6.000	11.000
*Ra*	2048	2.065	1.647	0.000	2.079	5.690
*Aa*	2048	1.678	1.342	0.000	1.609	4.331
*Size*	2048	21.963	1.147	18.516	21.788	26.284
*Lev*	2048	4.029	5.240	0.212	2.727	120.000
*Roa*	2048	0.042	0.108	−1.880	0.044	0.526
*Growth*	2048	0.454	4.218	−2.607	0.076	130.696
*Cash*	2048	0.069	0.096	−0.727	0.067	0.673
*Board*	2048	2.123	0.200	1.386	2.197	2.833
*Dual*	2048	0.302	0.459	0.000	0.000	1.000
*Share*	2048	0.357	0.147	0.040	0.349	0.960
*Ratio*	2048	0.372	0.062	0.143	0.333	0.800
*Age*	2048	2.132	0.936	0.000	2.398	3.434
*Soe*	2048	0.394	0.489	0.000	0.000	1.000
*Salary*	2048	15.088	0.909	11.472	15.068	18.293

### 4.2 Baseline Regression Results

The results of the benchmark regressions are reported in Table 2. Specifically, the estimation results in column (1) reveal that the regression coefficient of disclosure is significantly positive at the 1% level, thus indicating that such disclosure contributes to more government grants. The results in column (2) show that disclosure affects the intensity of government grants at the 5% significance level, which indicates that the higher the level of disclosure, the stronger the intensity of government subsidies; Hypothesis 1 is verified. This finding is consistent with the essence of disclosure theory, which is that disclosure has positive economic benefits and can significantly enhance a company's business performance and create economic value. The underlying mechanism is that disclosure by enterprises effectively reduces the degree of information asymmetry between corporate managers and the government and breaks down the information barriers between the two. In addition, the baseline regression results provide two insights. First, as a kind of quasi‐social responsibility information disclosure, such disclosure can effectively alleviate the problem of information asymmetry among multiple subjects. Second, the increase in the level of disclosure by firms can have positive economic effects.

**TABLE 2:** Analysis of baseline regression results.

Variable	*Sub*_1_	*Sub*_2_	3‐year term	5‐year term
	(1)	(2)	(3)	(4)
*Fsid*	0.110***	0.032**	0.022	−0.077
	(0.027)	(0.015)	(0.080)	(0.166)
*Size*	0.315***	0.618***	0.720***	0.845***
	(0.086)	(0.065)	(0.197)	(0.211)
*Lev*	−0.072***	−0.017***	−0.030	−0.034
	(0.015)	(0.005)	(0.021)	(0.022)
*Roa*	0.505	−0.418	2.992	3.219
	(1.121)	(0.385)	(2.704)	(2.664)
*Growth*	−0.466***	−0.076**	0.041	0.042
	(0.141)	(0.035)	(0.039)	(0.040)
*Cash*	−0.653	0.882**	−1.623	−2.054
	(1.061)	(0.404)	(2.147)	(2.172)
*Board*	−0.094	0.100	−1.006	−0.595
	(0.431)	(0.219)	(0.838)	(0.875)
*Dual*	−0.199	0.092	−0.794***	−0.914***
	(0.149)	(0.087)	(0.268)	(0.288)
*Share*	−3.332***	−1.139***	−6.531***	−7.612***
	(0.648)	(0.352)	(1.490)	(1.573)
*Ratio*	0.169	0.628	0.348	0.247
	(1.376)	(0.828)	(1.976)	(2.127)
*Age*	−0.787***	−0.005	−1.134***	−1.328***
	(0.101)	(0.041)	(0.244)	(0.271)
*Soe*	0.893***	0.082	0.352	0.398
	(0.177)	(0.088)	(0.311)	(0.332)
*Salary*	0.814***	0.205***	0.967***	0.948***
	(0.122)	(0.069)	(0.201)	(0.202)
*Region/Year*	Yes	Yes	Yes	Yes
*Constant*	−1.997	−15.554***	−9.390**	−11.333**
	(1.771)	(1.540)	(4.456)	(4.885)
*N*	2048	2048	508	485
*R‐squared*	0.164	0.211	0.238	0.259

*Note:* Robust standard errors are in parenthesis.

***p < 0.01; **p < 0.05.

Disclosure that is “inconsistent” is a form of pandering behavior that can help firms engage in low‐cost reputation building and demonstrate opportunistic behavior in their food safety production processes (Li et al. 2022). Hypothesis 1 confirms that voluntary food safety disclosure helps firms to obtain more grants. Then, two important and interesting questions arise: Is the speculative behavior adopted by some enterprises to obtain excessive government subsidies sustainable? Can the government identify and curb the moral hazard and opportunistic behavior of firms? Therefore, this part starts from this direction to explore the short‐term effect of “inconsistency,” which provides a theoretical basis and practical reference for regulators to identify firms' opportunistic behaviors. First, the median values of strategic philosophy and actual behavior in disclosure were used to filter out the sample firms that were “inconsistent.” That is, the sample of firms whose strategic philosophy disclosure is greater than the mean, and whose actual behavior is less than the mean (Bi et al. 2024). Second, disclosure was remeasured over a longer time window. With reference to relevant studies (Li et al. 2022), the weights were assigned to each year using the yearly product method (the central point of the method is that the closer the time, the greater the weight assigned, and the 3‐ and 5‐year term [−1,1], [−2,2] were selected), with the current year's weights being $n/\left(1+2+\ldots +n\right)$, and the other years' weights being $\left(n-t\right)/\left(1+2+\ldots +n\right)$, where *n* and *t* represent the window period time span and interval year, respectively. From the estimation results in columns (3) and (4) of Table 2, the coefficients on the 3‐ and 5‐year window are 0.022 and −0.077, respectively, both of which fail the significance test; thus, Hypothesis 2 is verified.

Specifically, firms may be selective and biased in their disclosure of nonfood safety information, thereby making it difficult for external stakeholders to obtain an accurate picture of the firm's food safety situation and effectively monitor its disclosure of food safety information. In this regard, the breadth and depth of the framework of food safety disclosure requirements by regulators should be improved. The results reveal that firms may exaggerate their food safety performance to send a signal to the outside world that their internal operating conditions are good, which may result in more grants in the short term; however, in the long term, such opportunistic disclosure behavior will not result in sustained gains, and government grants will decrease, which is not conducive to the sustainable development of firms and the shaping of their long‐term competitiveness.

### 4.3 Robustness Test

#### 4.3.1 Alternative Measures of Key Variables

Considering the impact of the choice of variable metrics and measures on the empirical findings, the explanatory and interpretive variables were remeasured, drawing on relevant research (Li et al. 2024). They are divided into 1–0 dummy variables according to their value, assigned a value of 1 above the mean value and 0 otherwise, and regressed anew. The estimation results of replacing the explanatory variable voluntary food safety disclosure (*Fsid diff*) are reported in column (1) of Table 3. The regression results are consistent with the baseline regression. Column (2) presents the estimation results of replacing the explanatory variable government grants (*Sub*_1_ *diff*), which is regressed using the Probit model since the explanatory variable is adjusted to be a dichotomous variable. The estimated coefficient of disclosure remains significantly positive at the 10% level, thus indicating that the estimates remain robust. In addition, the sign of the coefficients of the core variables, their significance, and the magnitude of the goodness‐of‐fit *R*_2_ between the different models did not change significantly when the measurement of the main variables was changed and the measurement method was altered. Furthermore, the above results support the significant positive relationship between disclosure and government grants, which confirms the robustness of our research conclusions.

**TABLE 3:** Robustness test results.

Variable	*Fsid diff*	*Sub*_1_ *diff*	PSM	2SLS‐IV	2008–2019
	(1)	(2)	(3)	(4)	(5)
*Fsid*	0.530***	0.021*	0.110***	0.395**	0.114***
	(0.132)	(0.013)	(0.027)	(0.164)	(0.031)
*Size*	0.322***	0.226***	0.304***	0.601***	0.306***
	(0.085)	(0.039)	(0.087)	(0.151)	(0.115)
*Lev*	−0.075***	−0.065***	−0.073***	−0.040**	−0.070***
	(0.016)	(0.009)	(0.015)	(0.016)	(0.019)
*Roa*	0.688	0.737*	0.662	1.538	−0.635
	(1.117)	(0.429)	(1.137)	(1.547)	(1.544)
*Growth*	−0.478***	−0.056	−0.464***	−0.038	−0.499***
	(0.142)	(0.035)	(0.141)	(0.023)	(0.161)
*Cash*	−0.680	−0.230	−0.653	−0.408	−1.505
	(1.058)	(0.408)	(1.061)	(1.085)	(1.325)
*Board*	−0.086	−0.169	−0.055	−0.419	−0.131
	(0.429)	(0.177)	(0.433)	(0.462)	(0.576)
*Dual*	−0.215	−0.100	−0.206	−0.011	−0.409**
	(0.150)	(0.071)	(0.150)	(0.159)	(0.191)
*Share*	−3.363***	−0.993***	−3.334***	−3.211***	−3.973***
	(0.647)	(0.243)	(0.648)	(0.665)	(0.797)
*Ratio*	0.184	−1.383**	0.217	−2.357	−0.482
	(1.362)	(0.568)	(1.378)	(1.825)	(1.695)
*Age*	−0.799***	−0.277***	−0.786***	−0.959***	−0.930***
	(0.101)	(0.044)	(0.101)	(0.157)	(0.135)
*Soe*	0.854***	0.226***	0.893***	0.567**	0.902***
	(0.177)	(0.076)	(0.177)	(0.255)	(0.208)
*Salary*	0.821***	0.311***	0.818***	0.741***	0.948***
	(0.122)	(0.045)	(0.122)	(0.141)	(0.148)
*Region/Year*	Yes	Yes	Yes	Yes	Yes
*Constant*	−1.435	−7.499***	−1.918	−1.553	−2.738
	(1.742)	(0.879)	(1.774)	(2.184)	(2.283)
*N*	2048	2048	2046	2048	1464
*R‐squared*	0.163	0.126	0.164	0.102	0.165

*Note:* Robust standard errors are in parenthesis.

***p < 0.01, **p < 0.05 and *p < 0.1.

#### 4.3.2 Propensity‐Score Matching

Propensity score matching (PSM) was used to mitigate possible sample selectivity bias and omitted variable problems. First, matching was performed using nearest‐neighbor matching to form the matched treatment and control groups. Second, the samples are matched in a 1:1 ratio, and the regressions are rerun. The results of the PSM test are reported in column (3) of Table 3. The regression coefficient of disclosure is positive and significant, which is consistent with the results of the benchmark regression test. This result suggests that the estimation of the benchmark regression is robust; that is, disclosure enhances government grants, and the conclusion of Hypothesis 1 still holds.

#### 4.3.3 2SLS Instrumental Variables Method

The instrumental variables approach was used to address the possible endogeneity of the research process. That is, whether some hidden factors jointly influence disclosure and government grants. Drawing on Wu et al. (2023), the mean value of food safety disclosure scores of other firms within the province where the firm is registered is selected as an instrumental variable. First, the rationale for selecting this is that the level of a firm's voluntary disclosure is correlated with that of other firms in the same province. That is, a cohort effect in information disclosure exists, and when firms in the same industry make such disclosure, other firms will imitate such behavior, and the two have a strong correlation. Second, theoretically, the level of food safety disclosure of other firms in the same province cannot have an impact on government grants, and no direct correlation exists between the two, which is an exogenous variable. The Kleibergen‐Paaprk Wald F‐statistic was used to test whether a weak identification problem exists with instrumental variables, and its statistic is 30.14, which is much larger than the critical value at the 10% significance level. This finding indicates that the level of food safety disclosure of other firms in the same province is not a weak instrumental variable for disclosure. The results of the relevant empirical tests are reported in column (4) of Table 3. Controlling for the original control variables and reestimating the baseline regression model using two‐stage least squares (2SLS) with instrumental variables (IV), disclosure still has a significant positive impact on government grants, and the coefficient estimates are more significant than the baseline regression coefficient estimates, thus suggesting that the previous regression results underestimated the impact of such disclosure on government grants.

#### 4.3.4 Excluding the Impact of Other Factors

Considering the impact of exogenous shocks on government grants caused by the outbreak of the COVID‐19 epidemic in 2020, it will cause some interference in the research findings. Therefore, this study reselects the sample interval, removes the data of 2020, 2021, and 2022, which are affected by the epidemic, and retests the sample of food firms in China's Shanghai and Shenzhen A‐shares from 2008 to 2019. The regression results in column (5) reveal that the conclusions remain robust after removing the impact of the epidemic.

## 5 Additional Tests

### 5.1 Moderating Effect of Market Attention

The economic consequences of voluntary food safety disclosure may exhibit both institutional and contextual dependence. As important information intermediaries in the capital market, analysts and research reports play an important role in the external monitoring environment. Voluntary food safety information for firms attracts more attention from securities analysts, especially in the Chinese capital market, where the regulatory system is weak and irrational investment behavior is high (Gao et al. 2021). Analysts' ratings, analysis, and interpretations of listed companies greatly enhance the transparency of firms' information, which helps other stakeholders better understand the actual status of firms and the adjustment of government grants. Referring to related research (Wang and Zhu 2022), two variables measuring market concern are used: the attention of the researched report and the analyst's attention, and the greater the attention of both proves that the market concern is more significant. As shown in column (2) of Table 4, the interaction between attention to the researched report and voluntary food safety disclosure is significantly negative, which indicates that the former has a significant negative moderating effect on the relationship between disclosure and government grants; that is, market attention weakens the economic effect of disclosure on government grants. In column (4) of Table 4, the coefficient of the interaction term between analyst attention and disclosure is negative and significant, thus indicating a negative moderating effect. Utilizing the mean value of the attention of the researched reports and the analysts' attention as a criterion, the areas above the mean value are classified as firms with high attention of the researched reports and high analysts' attention, while the ones that are not above the mean value are regarded as the firms with low attention of the researched reports and low analysts' attention. The specific results, as illustrated in columns (5–8) of Table 4, demonstrate that the positive impact of corporate voluntary food safety disclosure on government grants is diminished in firms with high attention. Market attention weakens the facilitating effect of such disclosure on government grants, and noninstitutionalized regulation can dampen the resource‐acquisition effect of disclosure. In summary, the possible explanation is that higher market attention inhibits the information effect. Analysts convey more private information about the firm's food safety status to the capital market and government agencies, ultimately adjusting the latter's evaluation of the firm's legitimacy and reducing government grants.

**TABLE 4:** Analysis of market attention results.

Variable	*Sub*_1_				Attention by research reports high	Attention by research reports low	Analyst attention high	Analyst attention low
	(1)	(2)	(3)	(4)	(5)	(6)	(7)	(8)
*Fsid*	0.110***	0.106***	0.111***	0.106***	0.057*	0.167***	0.047	0.153***
	(0.027)	(0.027)	(0.027)	(0.027)	(0.034)	(0.043)	(0.037)	(0.037)
*Ra*	−0.011	−0.004						
	(0.055)	(0.056)						
Ra*Fsid		−0.029*						
		(0.017)						
*Aa*			−0.034	−0.027				
			(0.069)	(0.070)				
Aa*Fsid				−0.038*				
				(0.021)				
*Controls*	Yes	Yes	Yes	Yes	Yes	Yes	Yes	Yes
*Region/Year*	Yes	Yes	Yes	Yes	Yes	Yes	Yes	Yes
*Constant*	−2.209	−1.659	−2.529	−1.969	1.143	1.624	1.694	1.682
	(2.076)	(2.100)	(2.089)	(2.113)	(2.429)	(2.638)	(2.772)	(2.172)
*N*	2048	2048	2048	2048	1149	899	900	1148
*R‐squared*	0.164	0.166	0.164	0.166	0.145	0.231	0.164	0.230

*Note:* Robust standard errors are in parenthesis.

***p < 0.01 and *p < 0.1.

### 5.2 Heterogeneity in Grant Types

What kind of government grants voluntary food safety disclosure can bring to firms is another important issue in this research. In the previous part of the theoretical analysis, food safety work was found to be an important part of local government performance appraisal, which affects government grants to a certain extent. Since access to capital is an important factor affecting firm growth, a reasonable assumption is that firms may adopt disclosure performance that is “inconsistent” to obtain more government grants or policy incentives to build up a good image of food safety. Government grants are its way of helping firms increase their relevant inputs. In the general sense, these grants include financial patent subsidies, training subsidies for firms, industrial support, high‐tech subsidies, credit preferences, and export earnings, among other types. In May 2017, the Ministry of Finance revised and issued the “Accounting Standard for Business Enterprises No. 16—Government Grants,” emphasizing that enterprises should truthfully disclose relevant information on government grants, including the amount, type, recognition basis, and conditions of government grants, in their financial reports in accordance with the requirements of the Accounting Standard for Business Enterprises. Concurrently, the government implements a range of measures to undertake continuous supervision and evaluation of enterprises receiving government grants. For instance, it meticulously scrutinizes the declaration qualifications and the authenticity of enterprises, fortifies the performance evaluation of government grant projects, and stipulates the earmarking of funds for designated purposes without any diversion.

According to the provisions of the current firm accounting standard, “government grants” can be categorized into revenue‐ and asset‐related. Revenue grants are mainly used for expenses or losses incurred or to be incurred by firms, with the core purpose of compensating for losses and cost‐sharing; asset grants refer to funds provided by the government to firms for the acquisition of fixed assets. A possible question is whether firms whose voluntary food safety disclosures are “inconsistent” are more interested in obtaining resources other than grants. Does the quality of such disclosure change firms' access to different types of grants? Do governments differentiate their grant policies for different disclosure categories of firms? Therefore, the delineation of government grants is critical to address the above questions. Unfortunately, the search of relevant databases and annual reports of firms reveals that only a small number of firms have provided a detailed description of the types of government grants, which makes it challenging to meet the requirements of the empirical analysis. In this regard, we briefly discuss a sample food firm as an example (Table 5). The firm's voluntary food safety disclosure is at a high level in the industry, and it obtained a total of 191,627,724.39 yuan of government grants in 2022. Compared with other food firms with the same level of sales, the value of revenue‐related grants is higher, and the difference with asset‐related government grants is not significant. The possible reason is that, on the one hand, income‐related government grants can make loss‐making firms turn around and solve the problem of short‐term financing constraints, which affects their current performance and then value creation ability. On the other hand, asset‐related government grants are mostly related to the procurement of fixed assets and other large‐scale equipment, which are cyclical and phased in nature, ultimately reflecting the dynamic adjustment of the reciprocal relationship between the government and enterprises more and thus continuously realizing the balance of interests between the government and firms.

**TABLE 5:** Analysis of government grants for a firm, in 2021–2022.

Project type	Amount in 2022	Amount in 2021	Code
1. Revenue‐related government grants			
(1) Fiscal discount grant	2260000.00	910000.00	[2009]31
(2) Financial grants	720000.00	7770000.00	[2008]165
(3) Write‐off of financial borrowings		2849600.00	[2009]425
(4) Financial liquidity grant	12170000.00		[2010]281
(5) Grants for harmless treatment of diseased pigs	2254040.00		[2009]52
(6) Agricultural industrialization subsidy	240000.00		[2010]202
(7) Incentives for promoting brand strategy	150000.00		[2009]10
(8) Others	300000.00	2467930.00	
Subtotal	18094040.00	13997530.00	
2. Asset‐related government grants			
(1) Postdisaster recovery and reconstruction fund	935351.06		[2008]3482
(2) Interest subsidy for meat processing projects	133333.33	133333.33	
Subtotal	1068684.39	133333.33	
Summation	19162724.39	14130863.33	

### 5.3 Heterogeneity in Firm Size

In reality, government grant acquisition is constrained not only by geographic location, economic development, and the institutional environment, but also by firm size. Whether larger or smaller firms receive more grants is an important topic given the differences in business conditions, profitability levels, and resource‐acquisition capabilities of firms of different sizes. Drawing on related research (Mao and Xu 2015), the sample is divided into two groups according to the median of the natural logarithm of the total assets of the firm at the end of the year, and those above the median are large‐scale firms, and vice versa for small‐scale firms. The regression results are reported in columns (1) and (2) of Table 6. The regression coefficient of the impact of disclosure on government grants in the large‐scale subgroup is 0.001, which is not significant. However, that in the subgroup of small‐scale firms is 0.167, which is significantly positive at the 1% level and passes the test of difference in coefficients between groups. Small‐scale firms have more advantages in disclosure and government grants compared to large‐scale firms. Significantly, the difference in the size of the firm determines the cost and benefit of information disclosure; small‐scale firms have relatively low operating profits, the government will pay more attention to such firms in the face of high‐quality disclosure, and government grants can be utilized to alleviate the financial constraints faced by small and medium‐sized enterprises (SMEs) to support the development of SMEs in the region.

**TABLE 6:** Analysis of heterogeneity results for firm size and city level.

Variable	*Sub*_1_				
	Large‐scale firms	Small‐scale firms	First‐line city	New first‐line city	Ordinary city
	(1)	(2)	(3)	(4)	(5)
*Fsid*	0.001	0.167***	0.034	−0.049*	0.156***
	(0.036)	(0.034)	(0.070)	(0.029)	(0.034)
*Controls*	Yes	Yes	Yes	Yes	Yes
*Region/Year*	Yes	Yes	Yes	Yes	Yes
*Constant*	5.322*	4.849**	2.787	−1.982	−1.192
	(2.720)	(2.224)	(5.087)	(2.638)	(2.119)
*N*	909	1139	311	248	1489
*R‐squared*	0.120	0.231	0.120	0.481	0.185

*Note:* Robust standard errors are in parenthesis.

***p < 0.01, **p < 0.05 and *p < 0.1.

### 5.4 Heterogeneity in City Levels

China's regional development imbalance has a long history of significant differences in each region's institutional environment and resource endowment. This external environment affects firm' decision‐making and strategic planning in a subtle but pervasive way (Fischer 2023). Whether and to what extent voluntary disclosure by firms can play a role is also closely related to the institutional environment of the region in which they are located. Therefore, referring to the “Business Attractiveness Ranking of Chinese Cities,” we group food firms according to the city level based on the location of the firm's headquarters, with the specific criteria of first‐tier, new first‐tier, and ordinary cities. The results of the regression of city‐level heterogeneity are reported in columns (3) to (5) of Table 6; the effect of first‐tier cities is not significant, while new first‐tier cities have a negative effect. However, the effect on ordinary cities is pronounced. The possible reason is that significant differences exist in firms' access to resources in different regions. Ordinary cities have a single channel through which firms can be publicized and presented to the outside world. Disclosure can effectively alleviate the information asymmetry and help firms win the trust of the government. By contrast, good government‐enterprise relations can help firms obtain the resources needed for their development, as well as stimulate the government to grant them subsidies. Therefore, voluntary food safety information disclosure can help ordinary urban firms obtain more external resources and promote high‐quality development.

## 6 Discussion and Implications

### 6.1 Discussion

Voluntary information disclosure is an important means of alleviating the degree of information asymmetry of stakeholders, which helps to transmit incremental information to the capital market and improve the information environment. Moreover, such disclosure is also an important scenario to optimize the rule system of China's mature capital market. However, little research has examined the resource‐acquisition effect of voluntary disclosure, especially the impact of voluntary food safety disclosure on government grants. Starting from the concept of the greater food approach, this study evaluates the impact of such disclosure on government grants using the annual data of Chinese‐listed food firms from 2008 to 2022. The findings of the study are as follows:

Firstly, our study found that voluntary food safety disclosure has a significant contribution to government grants and passes a number of robustness tests. The resource‐acquisition effect of voluntary food safety disclosure that is “not what it says on the tin” is only effective in the short term. Ultimately, the government's capacity to discern firms' pandering behavior is questionable, and such actions are ultimately not viable. Our findings extend the works of Pouliot and Wang (2018), as our study investigated the economic consequences of voluntary corporate disclosure. Based on the signaling theory and legitimacy theory, our study extends research on food safety disclosure (Chen et al. 2020; Fischer 2023; Lin, Xu, et al. 2024; Lin, Ma, et al. 2024). To a large extent, disclosure alleviates government pressure in traditional food safety regulations, helps to enhance government trust, and sends favorable signals to potential investors through policy orientation and regional support (Hribar et al. 2022; Bhattacharyya and Subrahmanya 2024). As government grants are a key resource and a kind of “credit certificate” provided by the government for firms, high‐quality disclosure meets the demands of both the government and firms (Seow 2024; Wei et al. 2024). However, certain firms have been observed to engage in the practice of exaggerating their food safety performance, with the intent of conveying to external observers an image of optimal internal operational effectiveness. In the short term, this strategy may assist firms in acquiring additional subsidies. From a long‐term perspective, such opportunistic disclosure behavior is ultimately untenable (Zhou et al. 2022; Hassanein and Albitar 2024). The validity of food safety disclosures is a critical concern that, once called into question, can result in a reduction of government subsidies. This, in turn, can have a detrimental effect on the realization of sustainable development and the long‐term competitiveness of enterprises.

Secondly, the mechanism of action test demonstrated that the coefficients of the interaction terms of market attention and analyst attention with voluntary food safety disclosure are negative and significant. This finding suggests that they negatively moderate the relationship between voluntary food safety disclosure and corporate subsidies. The market exerts a substantial influence on local governments, compelling them to regulate food safety information. Consequently, local governments place greater emphasis on the practical benefits of corporate food safety measures, thereby diminishing the positive correlation between “inconsistent” voluntary food safety disclosures and government subsidies. This finding is consistent with the conclusions of Lin, Xu, et al. 2024; Lin, Ma, et al. 2024 and Pope et al. (2024), who have demonstrated that external factors have a substantial impact on corporate behavior. In accordance with the principles of signaling theory, the heightened level of attention exhibited by higher analysts toward listed firms serves to mitigate the extent of information asymmetry, thereby facilitating a more comprehensive understanding of the risks and opportunities presented by these entities to external stakeholders. In the context of enterprises exhibiting speculative disclosure behavior, government subsidies may be subject to a gradual reduction or even complete elimination. In the future, the development of voluntary food safety disclosure normative guidelines should prioritize the comprehensiveness and accuracy of information disclosure, while also formulating detailed disclosure rules to prevent the exaggeration of favorable information. This would contribute to the enhancement of the food safety disclosure regulatory mechanism.

Thirdly, the results of the heterogeneity test demonstrate that the impact of voluntary food safety disclosure on corporate subsidies varies across types, regions, and firm sizes. First, with regard to the nature of subsidies, increased corporate subsidies from voluntary food safety disclosure are primarily concentrated in earnings‐related subsidies. A comparison of agro‐processing firms with analogous sales reveals that they receive substantially higher earnings‐related subsidies, while asset‐related subsidies demonstrate minimal variation. This finding aligns with the conclusions of Li et al. (2024), who reported that firms can obtain more earnings‐related subsidies through voluntary disclosure, leading to an improved financial position and enhanced short‐term performance. Secondly, from the perspective of the region to which it belongs, the information value of voluntary food safety disclosure is greater in the western region. In contrast, the effect of the first‐tier cities is not significant, and the new first‐tier cities even have a negative effect. Finally, the effect of the ordinary cities is very obvious. This finding suggests that the regional economic development level and policy environment significantly influence the relationship between food safety disclosure and corporate subsidies. Finally, in terms of firm size, small‐scale firms have more advantages in disclosure and corporate subsidies, and the size divide between firms is not obvious. This finding is consistent with the conclusions of Benameur et al. (2023), who posited that small‐scale firms are more adept at leveraging food safety disclosure to secure government subsidies due to their agility and capacity for innovation.

### 6.2 Practical Implications

Our findings have several practical implications. Firstly, for policymakers: Thick food safety governance, establish the greater food approach and digital information concept. Voluntary food safety information disclosure significantly increases the level of government grants, and the core mechanism is the information resource effect, which reduces information and communication barriers. Therefore, in the context of “the greater food approach” and other major national needs, full use should be made of the incentive mechanism of quality food safety information, the concept of “digital information” established, and a food safety governance system that adapts to the development needs of the data era constructed. At the same time, we should rely on the seamless interaction of multiple interest groups to participate in food information supervision and form a three‐dimensional vertical and horizontal information governance structure.

Secondly, for food companies: The information disclosure system arrangement should be improved and a long‐term mechanism for food safety information disclosure built. The short‐term effect of the “inconsistent” behavior shows that government grants can be used to guide firms to improve their food safety awareness and management level, but the formation of a long‐term promotion mechanism is difficult. The government should conduct in‐depth research on the promotion mechanism and basic institutional arrangements that combine short‐ and long‐term food safety information disclosure, adhere to the top‐level design leadership, and continue to stimulate the endogenous motivation of firms to improve food safety, so as to form a favorable situation of reducing food safety incidents from the root. In response to the manipulation of disclosure and “inconsistent” behavior in disclosure, the new mechanism of ex ante selection, ex post supervision, and ex post evaluation should be improved so as to promote the orderly development of food safety information disclosure.

Thirdly, for regulatory bodies: The multibody, multicenter collaborative governance model for the food information chain must be established. The test of the role mechanism reveals that noninstitutional regulation provides an important tool for the government to realize information governance, and to build a food information data monitoring platform with the synergy of multiple subjects is important. In this regard, the government, consumers, investment institutions, social organizations, and other subjects need to cooperate in information governance and to establish a multisubject, multilevel, multidimensional information governance mechanism. For food safety, a “whistleblower” system requires positive reports and other signaling mechanisms to achieve the external incentives for food safety substantive behavior. Ultimately, the formation of substantive behavior continues to induce the manipulation of timely exposure to the food information governance model. The responsibility of food safety supervision must be implemented, and the performance appraisal system of local food safety governments must be deepened. The degree of attention to food information in the actual assessment should be enhanced, regional food safety risks as the core objective of the assessment controlled, and the benign cycle of food safety information regulation realized.

### 6.3 Limitations and Recommendation

While our research advances the literature, it is not without limitations, which serve as opportunities for future research. Firstly, the study's primary focus on China introduces limitations in terms of generalizability to other regions characterized by distinct regulatory and market environments. Secondly, the study is predominantly supported by annual reports and official data, which may not fully capture the intricacies of corporate behavior and information manipulation. To further refine our understanding of voluntary food safety disclosure strategies and their impact on food safety governance and resource allocation, future research should consider cross‐country comparisons, longitudinal analyses, and alternative data sources. Finally, the collection process of gathering voluntary food safety disclosure indicators from annual corporate and social responsibility reports needs to be optimized. In the future, academic research endeavors may employ Python big data crawler technology to collect and obtain data from unlisted companies. These studies can then be used to empirically test the theoretical research, thereby providing empirical evidence regarding voluntary food safety disclosure variable measures and dynamic effects.

## Author Contributions

**Wentai Bi:** resources (equal), writing – review and editing (equal). **Jie Huan:** conceptualization (equal), funding acquisition (equal). **Peihang Zhang:** investigation (equal), visualization (equal). **Xudong Guo:** software (equal), visualization (equal). **Qi Qi:** data curation (equal), methodology (equal). **Yuan Liang:** methodology (equal), validation (equal).

## Conflicts of Interest

The authors declare no conflicts of interest.

## Data Availability

The data that support the findings of this study are available from the corresponding author upon reasonable request.

## Appendix 1 Raw Food Safety Disclosure Score Summary Statistics in 2008 and 2022

**Table:**

Disclosure category	Primary indicators	Secondary indicators	2008	2011	2014	2017	2020	2022
Strategic concept (Soft disclosure items)	Vision and strategy statement	Statement of food quality and safety policies, values, principles and behaviors	0.409	0.643	0.681	0.795	0.768	0.867
		Statements to prevent food quality and safety risks	0.057	0.198	0.206	0.375	0.389	0.436
		Statement of preparation for the development of new food technology	0.545	1.262	1.241	0.784	0.941	1.058
		Statement of the company's food quality compared to industry peers	0.489	0.865	0.787	0.642	0.576	0.587
	Corporate image	Separate social responsibility reports	0.455	0.698	0.702	0.847	0.857	0.916
		Participate in food quality and safety activities or meetings	0.148	0.087	0.057	0.188	0.069	0.298
		Participate in food safety improvement activities organized by industry associations or on own initiative	0.273	0.508	0.546	0.301	0.340	0.133
Actual behavior (Hard disclosure items)	Governance structure and management	Establishing a risk management committee in the governance organization	0.102	0.190	0.177	0.364	0.739	0.720
		Set up food quality control department or quality management department	0.198	0.190	0.241	0.551	0.936	1.071
	Control over suppliers and sellers	Establishing supplier‐specific food quality control measures	0.455	0.675	0.738	0.676	0.862	0.840
		Establishment of food quality control measures for sellers	0.545	0.238	0.277	0.511	0.557	0.747
	Control of production process	Food safety certification	0.318	0.429	0.496	0.574	0.724	0.787
		Establishing a prevention and control system for food safety risks	0.114	0.325	0.369	0.364	0.468	0.404
		To establish the whole process of food quality monitoring and traceability system	0.159	0.405	0.461	0.523	0.330	0.280
		Technology and R&D costs to improve food safety	0.761	0.373	0.447	0.483	0.901	0.942
		Expression of food quality qualification rate	0.182	0.214	0.312	0.398	0.626	0.573
		Presence of food safety warning and accident response programs	0.148	0.770	0.723	0.614	0.251	0.249
	Control of employee behavior	Training for employees on food quality management and operation	0.386	0.762	0.787	0.506	0.783	0.942
		Food safety performance incentives for employees	0.284	0.246	0.277	0.369	0.335	0.289

*Note:* 2008–2022, % of firms attaining item.

## Appendix 2 Variable Definitions

**Table:**

Variable	Abbreviations	Measurement
Government grant level	*Sub*_1_	Amount of government grants received by the firm during the year +1 to take logarithmic values
Intensity of government grants	*Sub*_2_	(Total government grants to firms/industry average grant level) +1
Voluntary food safety disclosure	*Fsid*	Calculation of indicators for evaluating the quality of voluntary food safety disclosures
Strategic concept	*Stra*	Calculation of indicators for evaluating the quality of strategic concept
Actual behavior	*Beha*	Calculation of indicators for evaluating the quality of actual behavior
Research reported attention	*Ra*	Number of research papers tracked and analyzed by firms +1 takes logarithmic value
Analyst Attention	*Aa*	Number of analysts tracked +1 takes logarithm
Firm size	*Size*	Ln (Total assets)
Leverage ratio	*Lev*	Total liabilities/Total assets
Return on assets	*Roa*	Reported net profit after tax/Total assets
Revenue growth rate	*Growth*	(Current year revenue–Past year revenue)/Past year revenue
Operating cash flow	*Cash*	Cash flow from operating activities/total assets
Board size	*Board*	Logarithmic total number of board members
Dual position	*Dual*	An indicator that equals 1 when the chairman of the firm takes the position of CEO, and 0 otherwise
Shareholding concentration	*Share*	Shareholding ratio of the largest shareholder
Proportion of independent directors	*Ratio*	Number of independent directors/total number of board members
Fixed number of years	*Age*	Ln (Number of years since the establishment of the firm +1)
Nature of ownership	*Soe*	The ultimate controller is the State, which takes the value of 1, otherwise it is 0
Executive remuneration	*Salary*	Ln (Top 3 executives' remuneration)
